# Association of taurine intake with changes in physical fitness among community-dwelling middle-aged and older Japanese adults: an 8-year longitudinal study

**DOI:** 10.3389/fnut.2024.1337738

**Published:** 2024-03-20

**Authors:** Takashi Domoto, Kazuyoshi Kise, Yukiko Oyama, Kanae Furuya, Yuki Kato, Yukiko Nishita, Rumi Kozakai, Rei Otsuka

**Affiliations:** ^1^Self-Medication Research and Development Headquarters, Taisho Pharmaceutical Co., Ltd., Tokyo, Japan; ^2^Department of Epidemiology of Aging, Research Institute, National Center for Geriatrics and Gerontology, Obu, Japan; ^3^Faculty of Health and Medical Sciences, Aichi Shukutoku University, Nagakute, Japan; ^4^School of Lifelong Sport, Hokusho University, Ebetsu, Japan

**Keywords:** taurine, dairy intake, muscle strength, older people, longitudinal study

## Abstract

**Introduction:**

Taurine has diverse valuable biological functions, including antioxidant activity and regulation of osmotic pressure. Maintaining physical fitness from middle age is important for healthy life expectancy. Although taurine administration improves muscle endurance and strength, its role in maintenance remains unclear. We aimed to clarify the longitudinal taurine intake association with fitness changes.

**Methods:**

Participants comprised men and women aged ≥40 years who participated in the third (2002–2004; Baseline) and seventh (2010–2012; Follow-up) waves of the National Institute for Longevity Sciences-Longitudinal Study of Aging (NILS-LSA) and completed a 3-day dietary weights recording survey at baseline. A table of taurine content was prepared for 751 foods (including five food groups: Seaweed; Fish and shellfish; Meat; Eggs; and Milk and dairy products) from the Standard Tables of Food Composition in Japan (1,878 foods) 2010. Four physical fitness items (knee extension muscle strength, sit-and-reach, one-leg standing with eyes closed, and maximum walking speed) were measured at baseline and follow-up. We analyzed the association of taurine intake with physical fitness change, employing a general linear model (GLM) and trend tests for baseline taurine intake and follow-up fitness change. Adjustments included baseline variables: sex, age, height, weight, educational level, self-rated health, smoking status, depressive symptoms, and clinical history.

**Results:**

The estimated average daily taurine intake (standard deviation) was 207.5 (145.6) mg/day at the baseline. When examining the association with the four physical fitness parameters, higher taurine intake positively increased the change in knee extension muscle strength (T1; 0.1, T2; 0.8, T3; 1.1 (kgf) GLM, *p* < 0.05; p for trend <0.05) and reduced the decline in knee extension muscle strength in the subgroup analysis of participants aged ≥65 years (T1: −1.9, T2: −1.7, T3: −0.4 kgf; GLM *p* < 0.05, p for trend <0.05). No relationship was found between taurine intake and the remaining three fitness factors.

**Conclusion:**

Estimation of taurine intake showed that dietary taurine intake potentially contributes to the maintenance of knee extension muscle strength over 8 years among Japanese community-dwelling middle-aged and older individuals. This is the first study to investigate the association of dietary taurine intake with muscle strength.

## 1 Introduction

Taurine, an amino sulfonic acid, has various useful functions *in vivo*, such as antioxidant activity, osmotic regulation, and bile acid conjugation (1). A recent report (2) indicated that human blood taurine levels decrease with age. In mice, taurine administration improved coordination, muscle endurance, muscle strength, and prolonged lifespan. Furthermore, taurine improved the health-related indicators of monkeys and extended their healthy lifespan. Moreover, the same study reported that taurine administration delayed the deterioration of aging-related biomarkers in mice and zebrafish. Taurine is found in food, mainly fish and shellfish, and dietary taurine intake has been reported in South Korea (3–5). Taurine improves athletic endurance (6) and muscle performance by regulating the release of calcium ions from muscle cells (7).

Physical fitness is defined as the ability to perform daily activities with optimal performance, endurance, and strength while managing disease, fatigue, stress, and reduced sedentary behavior (8). People with reduced physical fitness face many problems, including increased healthcare costs, sudden cardiac death, and increased mortality (9–12). For example, maintaining activities of daily living (ADL) is important for older adults to be able to live independently. Furthermore, as physical fitness declines, ADLs may decline. Consequently, individuals may become bedridden owing to their reduced activity. Therefore, maintaining physical fitness as much as possible from middle age is important to extend a healthy life expectancy. A relationship between physical fitness and diet-derived nutrients has also been reported (13). However, to the best of our knowledge, there are few reports on the relationship between dietary taurine intake and physical fitness.

Thus, this study aimed to clarify the longitudinal association between dietary intake of taurine and changes in physical fitness in middle-aged and older adults living in the community, which has not been reported so far in Japan.

## 2 Materials and methods

### 2.1 Participants

**Figure 1:**
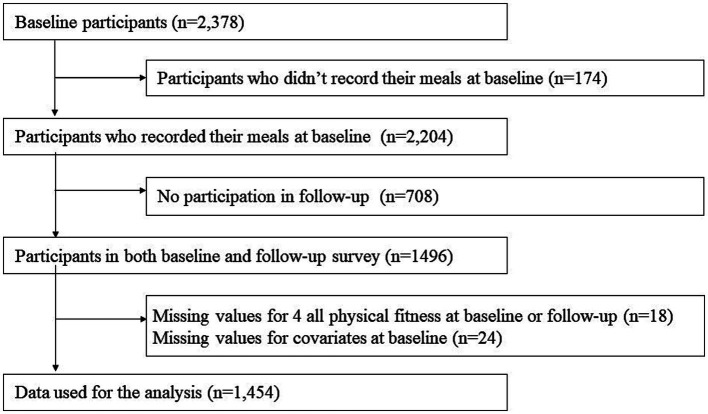
Flowchart depicting participant selection and disposition.

Data for this study were obtained from the National Institute for Longevity Sciences, Longitudinal Study of Aging (NILS-LSA), which used detailed questionnaires, medical checkups, anthropometric measurements, physical fitness tests, and nutritional examinations to assess the normal aging process over time. The first wave of the NILS-LSA was conducted from November 1997 to April 2000 and included 2,267 participants (1,139 men and 1,128 women; age range: 40–79 years). A follow-up survey was conducted every 2 to 5 years. Participants (except those aged >79) who could not attend follow-up investigations were replaced with new, randomly recruited age-and sex-matched participants. Participants aged 40 years were recruited annually. The details of the NILS-LSA have been reported previously (14). Figure 1 shows the number of participants included in the analysis. Participants were selected from a baseline study (*n* = 2,378). The exclusion criteria were as follows: (i) individuals who did not record their meals at baseline (*n* = 174), (ii) those who did not participate in the follow-up (*n* = 708), and (iii) individuals who lacked values for all four physical fitness measures (*n* = 18) and covariates (*n* = 24). Finally, 1,454 participants were included in the analysis. The individual numbers, excluding those with deficiencies in dietary and physical fitness data, were as follows: knee extension muscle strength: 1,254; sit-and-reach: 1,426; closed-eyed one-legged standing: 1,413; maximum walking speed: 1,385. After approval by the Ethics Committee of the National Institute for Longevity Sciences (NILS-LSA), a briefing session was held to provide study-related information to the participants, and those who gave their written informed consent were included in the study. This study on human participants was reviewed and approved by the Ethics Committees of the National Institute for Longevity Sciences and Taisho Pharmaceutical Co., Ltd.

### 2.2 Preparation of a provisional taurine content table

In this study, a provisional content table was prepared according to the procedure described in Supplementary Table S1. The taurine content data was collected for 751 food items (Seaweed, Fish and shellfish, Meat, Eggs, and Milk and dairy products) from the 1,878 food items listed in the Standard Tables of Food Composition in Japan 2010 using the following information sources. According to the methods described below, a provisional content table for 666 (87.3%) of the 751 target foods was prepared (Supplementary Table S1).

#### 2.2.1 Citations from academic papers, other sources, and new measurements

Based on a review of academic papers, other sources, and new measurements, a total of 197 food items were selected. Among them, the taurine contents of 54, 121, and 14 foods were selected from the “Table of Free Amino Acid Content of Foods (15)” (Japan Society of Nutrition and Food Science), academic papers (16–22), and the “National Standard Food Composition Table” of the Korean Rural Development Agency (23), respectively. The taurine content of eight foods was measured by Japan Food Research Laboratories. In cases where multiple values were available from the cited sources, the average of these values was used. Unless otherwise specified, values for raw fish and meat items (and even higher values for fatty meat items) were used. Furthermore, if the protein content was stated in the source material, it was corrected according to the weight by using the protein content in the Standard Tables of Food Composition of Japan 2010 (24).

#### 2.2.2 Complement of taurine content to similar foods

For foods that could not be cited using the method described in subsection 2.2.1, the following methods were used to supplement the data. The same foods with different components were complemented with the data of the most similar part (e.g., “Japanese beef, thigh meat, no subcutaneous fat - raw” was complemented with “Japanese beef, thigh meat, red meat - raw”). In the case of identical species, the value of the most similar species was used for data completion (e.g., “Shiba shrimp - raw” was completed using the value of “Japanese tiger prawn - cultured - raw,” which is also one of the species of the Penaeidae family). Values of dried products, such as seaweed, which were rehydrated in water, were corrected for moisture. When complementing the taurine content of beef and fish that were similar to closely related species and different parts, the content was corrected according to the protein content. Milk/egg-processed products (three processed egg products: egg tofu, thick-boiled eggs, and Japanese omelets were calculated based on the proportion of eggs in the food composition table) were also corrected according to the protein content. Changes in the content of cooked products such as “boiled” and “grilled” fish and meat were not considered (as the quantitative impact is unclear). For the four products with a protein content of zero in the Standard Tables of Composition of Japan (e.g., vegetable cream and other products classified as “milk and dairy products”), the taurine content was set to 0. Including these items, there were 85 out of 751 products for which no content was set; however, these products were consumed by 25 or fewer people (42 of these products were consumed by none of the participants).

#### 2.2.3 Nutritional assessments and calculation of estimated taurine and nutrient intakes

The details of the nutritional assessments conducted as part of the original survey have been previously reported (25). The participants were instructed to undertake the survey on days when they ate as many normal meals as possible, avoiding special days, such as annual days, because the dietary survey was conducted to assess their normal dietary habits. During this time, a 1-kg kitchen scale (Sekisui Jushi, Tokyo, Japan) and one or more disposable cameras (27 shots; Fuji Film, Tokyo, Japan) were provided to all participants, and a practice session on taking pictures with the disposable camera was conducted. After participation in the baseline study, participants completed a 3-day dietary record to assess their dietary intake, including the use of supplements. The dietary record was completed over three consecutive days (two weekdays and one weekend day) because food habits differed on weekdays and weekends; three consecutive days were selected based on the same method used by the National Health and Nutrition Examination Survey in Japan. Before cooking, all food items, including spices and seasonings, were placed in a lightweight cup or spoon and weighed or measured separately on a 1-kg kitchen scale, or the portion sizes were estimated. During the 3 days of the survey, detailed notes of all eating and drinking activities, including snack consumption, were recorded. Furthermore, the participants used a disposable camera to photograph their meals, both before and after eating. Participants completed the dietary record at home, and most returned it within 1 month. The films were developed using returned disposable cameras. Dietitians then used these photos to complete the missing information in each participant’s dietary record and assigned a code number to every food from the Standard Tables of Food Composition in Japan 2010. Taurine intake was estimated by adding the amount of food consumed to provisional taurine content tables. The average daily dietary taurine intake (mg/day; not including supplements, drugs, and quasi-drugs) was estimated by analyzing the results of the nutritional assessments against the provisional taurine content table.

### 2.3 Physical fitness measurements

We selected four physical fitness items as the typical elements: knee extension muscle strength (muscle strength), sit-and-reach (flexibility), one-leg standing with eyes closed (balance), and maximum walking speed (mobility). Details of these physical fitness measurements have been reported previously (26, 27).

Knee extension muscle strength was measured with a measurement device (T.K.K.1281a, Takei Scientific Instruments Co., Niigata, Japan) with the participant seated upright and the knee and hip flexed at 90°, following the routine practice in physical fitness studies. Measurements of knee extension strength were repeated three times, and the maximum values were recorded. The average value of the measurements for the right and left knees was used for the analysis.

The sit-and-reach test was performed with a standard box with a scale on the top (T.K.K.4308a, Takei Scientific Instruments Co., Niigata, Japan). The participants were required to sit with both legs outstretched on the floor and to reach forward as far as possible while pushing a slide over the box with their fingertips.

The one-leg standing test was performed with eyes closed and measured the time spent standing on one leg with eyes closed, legs bent, and hands on the hips. Participants selected the leg they would use for the standing task. A stopwatch was used to record the time taken to lower the leg, change the position of the standing leg, open their eyes, and remove their hands from the waist. The longer duration of the two trials was used in the analysis.

The maximum walking speed was measured using the gait analysis system YW3 (Yagami Corporation). A space of approximately 1 m was provided, both before and after the test distance, for acceleration and deceleration, and participants walked 10 m in comfortable walking shoes. To measure maximum speed, the participants were instructed to walk as fast as possible without running. Optical sensors were used at the start and endpoint, and the time taken to walk 10 m was recorded.

During all fitness measurements, the safety of the participants was ascertained thoroughly. The examiner carefully measured physical fitness while monitoring the participants’ blood pressure and fatigue levels. Before starting the physical fitness measurements, a doctor asked the participants about their health status. Participants with serious physical or mental problems or those with any orthopedic or cardiovascular concerns were excluded from the measurements.

### 2.4 Statistical analysis

All statistical analyses were conducted using SAS/STAT 9.3_M1 (SAS Institute Japan Ltd.). A general linear model (GLM) and trend test were used to assess the associations between various items. Trend associations were assessed by utilizing dummy variables (−1, 0, and 1) that were assigned to the dietary taurine intake tertiles. The association of baseline dietary taurine intake with the intake of various food groups was assessed. Next, the association of baseline dietary taurine intake with changes (baseline to follow-up) in four fitness items (knee extension muscle strength, sit-and-reach, closed-eye one-leg standing, and maximum walking speed) was assessed. Model 1 was adjusted for baseline sex, age, height, weight, educational level, self-rated health, smoking status, depressive symptoms, and clinical history (hypertension, heart disease, stroke, dyslipidemia, and diabetes). In the GLM, the baseline dietary taurine intake was included as a covariate, whereas, in the trend test, the group (tertile of dietary taurine intake) was added as a covariate. Model 2 was further adjusted for baseline physical fitness, with a subgroup analysis performed separately for participants aged 40–65 years and 65 years and older. The significance level was set at *p* < 0.05.

## 3 Results

Table 1 shows the baseline characteristics of the participants. The proportion of men to women was almost 50/50; as only participants older than 40 years were enrolled, the average age was 56.8 years. The estimated mean daily taurine intake (standard deviation) at baseline was 207.5 (145.6) mg/day. Table 2 presents food intake according to the taurine intake at baseline. The average intake (standard deviation) of each tertile of taurine intake was as follows: T1: 89.1 ± 28.1; T2: 170.9 ± 25.4; and T3: 362.6 ± 151.2 mg/day. Furthermore, taurine intake was significantly associated not only with Seaweed, Fish, and shellfish but also with the intake of cereals, potatoes, non-green yellow vegetables, mushrooms, and total energy intake.

**Table 1:** Baseline characteristics of all the participants.

Baseline characteristics of all the participants (*n* = 1,454)				Values	
Men, % (*n*)				49.4 (719)	
Age [years] (Mean ± SD)				56.8 ± 10.3	
Height [cm] (Mean ± SD)				160.1 ± 8.8	
Weight [kg] (Mean ± SD)				59.0 ± 10.2	
Education [Years] (Mean ± SD)				12.6 ± 2.7	
Self-rated health, % (*n*)					
	Excellent/very good			30.1 (438)	
	Good			62.5 (909)	
	Fair/Poor			7.4 (107)	
Smoking status, % (*n*)					
	Never			58.3 (847)	
	Former			23.8 (346)	
	Current			18.0 (261)	
Clinical history, % (*n*)					
	Hypertension			22.8 (332)	
	Heart disease			3.6 (52)	
	Stroke			1.7 (25)	
	Dyslipidemia			17.9 (260)	
	Diabetes			6.1 (89)	
Depressive symptoms, % (*n*)					
	CES-D (>16 points)			10.5 (152)	
Knee extension muscle strength [kgf] (Mean ± SD)		*n* = 1254^a^			34.2 ± 11.0
sit-and-reach [cm] (Mean ± SD)		*n* = 1426^b^			6.1 ± 9.1
One-leg standing with the closed eye [s] (Mean ± SD)		*n* = 1413^c^			19.6 ± 27.2
Maximum walking speed [m/min] (Mean ± SD)		*n* = 1385^d^			108.9 ± 12.5
Energy [kcal/day] (Mean ± SD)					2161.7 ± 415.4
Taurine [mg/day] (Mean ± SD)					207.5 ± 145.6

a, missing value for baseline or follow-up data (*n* = 200); b, missing value for baseline or follow-up data (*n* = 28); c, missing value for baseline or follow-up data (*n* = 41); d, missing value for baseline or follow-up data (*n* = 69).

**Table 2:** Food intake stratified according to the taurine intake at baseline.

	Tertiles of taurine intake (Mea*n* ± SD)			Value of *p*^†^	Trend value of *p*^‡^
	T1 (Low) 92.3*(*n* = 484)	T2 (Middle) 168.2*(*n* = 486)	T3 (High) 321.5*(*n* = 484)		
Cereals (g/day)	450.2 ± 135.1	461.1 ± 128.1	477.7 ± 144.4	0.007	0.002
Potatoes (g/day)	41.8 ± 34.0	46.5 ± 33.7	46.5 ± 33.4	0.042	0.029
Beans (g/day)	65.0 ± 80.6	70.3 ± 51.3	67.8 ± 54.5	0.425	0.486
Nuts and seeds (g/day)	3.7 ± 6.2	4.2 ± 7.8	3.8 ± 6.1	0.477	0.761
Green yellow vegetables (g/day)	105.2 ± 65.8	112.6 ± 67.1	114.4 ± 68.4	0.075	0.032
Non-green yellow vegetables (g/day)	176.5 ± 80.0	196.8 ± 90.2	214.2 ± 94.5	<0.001	<0.001
Fruits (g/day)	134.1 ± 112.6	155.4 ± 126.8	143.7 ± 126.0	0.025	0.225
Mushrooms (g/day)	10.7 ± 12.1	12.3 ± 13.6	14.1 ± 16.1	0.001	<0.001
Seaweed (g/day)	15.4 ± 17.3	17.8 ± 18.6	20.3 ± 21.8	<0.001	<0.001
Fish and shellfish (g/day)	60.0 ± 32.7	94.6 ± 37.6	143.7 ± 56.5	<0.001	<0.001
Meats (g/day)	63.0 ± 39.4	68.6 ± 40.9	69.1 ± 42.0	0.037	0.021
Eggs (g/day)	44.2 ± 24.7	44.8 ± 24.8	47.6 ± 27.4	0.079	0.035
Milk and dairy products (g/day)	160.3 ± 126.6	167.6 ± 135.0	145.5 ± 126.2	0.026	0.076
Energy [kcal/day]	2030.9 ± 388.2	2158.9 ± 392.1	2295.5 ± 423.0	<0.001	<0.001
Taurine [mg/day]	89.1 ± 28.1	170.9 ± 25.4	362.6 ± 151.2	<0.001	<0.001

*Median of taurine intake (mg/day).

^†^GLM, the analysis of variance was used.

^‡^Trend test, a general linear model was used to assign dummy variables (−1, 0, and 1) to the tertiles of dietary taurine intake.

SD, standard deviation.

**Figure 2:**
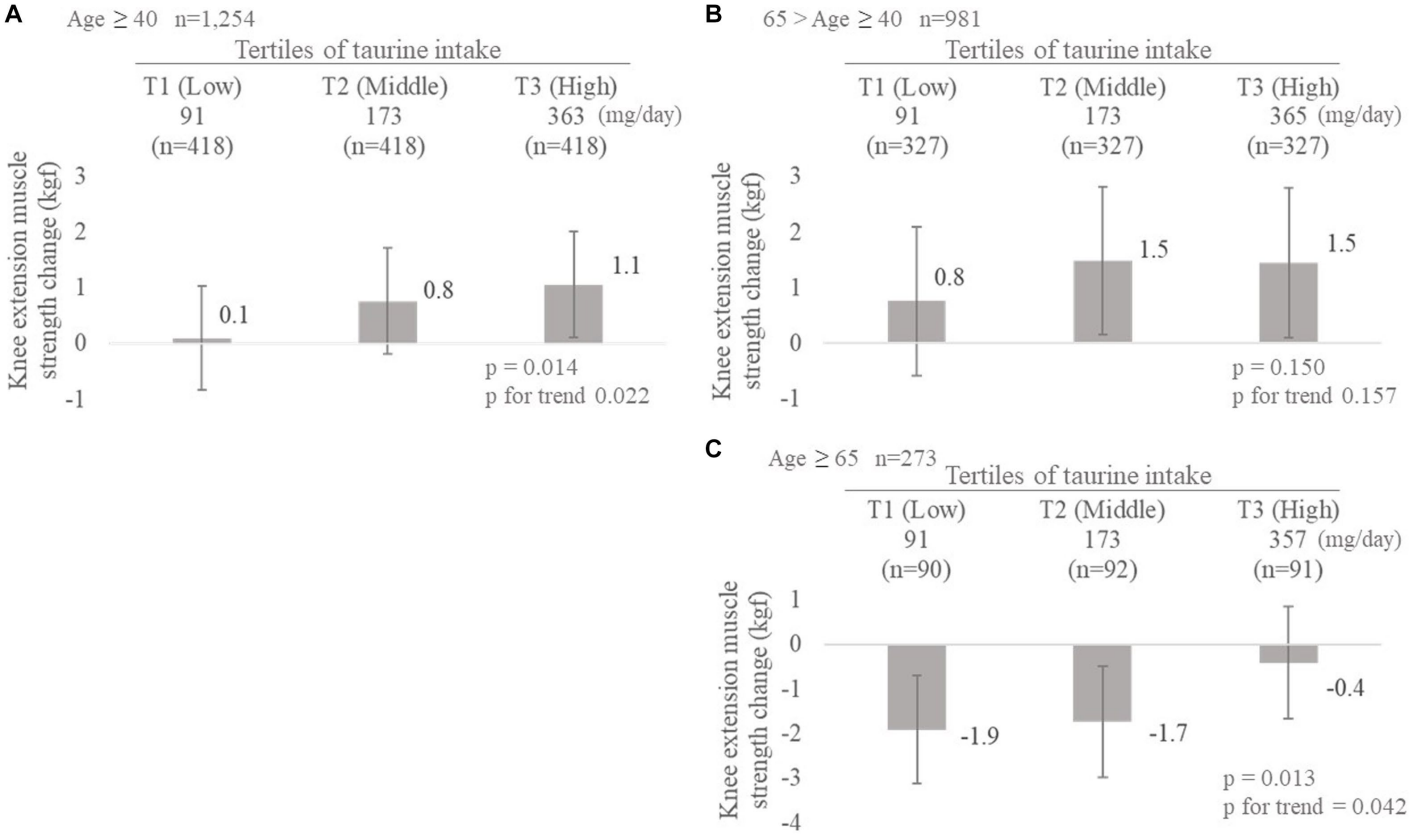
Effect of taurine intake on changes in knee extension muscle strength. **(A)** All participants (*n* = 1,254). **(B)** Participants aged 40–64 years (*n* = 981). **(C)** Participants over 65 years old (*n* = 273) mean ± SD.

Table 3 shows each physical fitness according to tertiles of taurine intake at baseline. Taurine was associated with the change from baseline of only knee extension muscle strength (GLM *p* < 0.05, *p* for trend <0.05). This trend did not change after adjustment with the baseline physical fitness parameters (Model 2). No relationship was found between taurine intake and the remaining three fitness factors. The results from Model 2 for the knee extension muscle strength have been shown as a graph in Figure 2A. Based on the evaluation of the tertiles of taurine intake (T1: low, T2: medium, and T3: high), high taurine intake maintained knee extensor muscle strength (T1; 0.1, T2; 0.8, and T3; 1.1 kgf; GLM *p* < 0.05, p for trend <0.05). Furthermore, this trend was observed in participants aged 65 years or more, where higher taurine intake prevented a reduction in muscle strength (T1: −1.9, T2: −1.7, T3: −0.4 kgf; GLM *p* < 0.05, p for trend <0.05; Figure 2C).

**Table 3:** Multivariate analysis of the association between taurine intake and physical fitness.

			Tertiles of taurine intake (Mean ± SD)			Value of *p*^†^	Trend value of *p*^‡^
			T1 (Low)	T2 (Middle)	T3 (High)		
Knee extension Muscle strength [kgf]			*n* = 418	*n* = 418	*n* = 418		
	Baseline	Model 1	29.7 ± 1.1	30.2 ± 1.1	30.2 ± 1.1	0.511	0.306
	Change from baseline	Model 1	1.0 ± 1.0	1.5 ± 1.0	1.8 ± 1.0	0.023*	0.044*
		Model 2	0.1 ± 0.9	0.8 ± 0.9	1.1 ± 1.0	0.014*	0.022*
Sit-and-reach [cm]			*n* = 476	*n* = 474	*n* = 476		
	Baseline	Model 1	6.0 ± 1.1	6.3 ± 1.1	6.6 ± 1.1	0.378	0.244
	Change from baseline	Model 1	−2.0 ± 0.6	−1.8 ± 0.6	−1.7 ± 0.6	0.148	0.351
		Model 2	−2.0 ± 0.6	−1.8 ± 0.6	−1.7 ± 0.6	0.087	0.211
One-leg standingWith eyes closed [s]			*n* = 471	*n* = 471	*n* = 471		
	Baseline	Model 1	13.8 ± 3.5	14.0 ± 3.6	13.3 ± 3.6	0.920	0.738
	Change from baseline	Model 1	−3.7 ± 3.3	−3.8 ± 3.3	−3.1 ± 3.3	0.663	0.651
		Model 2	−7.4 ± 2.4	−7.4 ± 2.4	−7.1±2.4	0.614	0.759
Maximum walkingSpeed [m/min]			*n* = 461	*n* = 462	*n* = 462		
	Baseline	Model 1	104.9 ± 1.6	106.1 ± 1.6	106.5 ± 1.6	0.059	0.036
	Change from baseline	Model 1	−1.5 ± 1.5	−2.9 ± 1.5	−2.5 ± 1.5	0.919	0.143
		Model 2	−2.9 ± 1.4	−3.9±1.4	−3.3 ± 1.4	0.373	0.472

^†^GLM, general linear model used.

^‡^Trend test, the general linear model was used by assigning dummy variables (−1, 0, and 1) to the tertiles of dietary taurine intake.

Model 1: Baseline sex, age, weight, height, smoking status, self-rated health, education, and clinical history of hypertension, heart disease, stroke, dyslipidemia, diabetes, and depressive symptoms. GLM: + bacseline dietary taurine intake. Trend test: + group (tertiles of dietary taurine intake).

Model 2: Model 1 + baseline values of each physical fitness parameter. The change from baseline was calculated as: “follow-up” − “baseline.”

^*^Significance level at *p* < 0.05.

## 4 Discussion

This study represents the first investigation into the association between dietary taurine intake and muscle strength. The findings from this retrospective study indicate that taurine intake is positively associated with knee extension muscle strength but not with the other three fitness factors (sit-and-reach, one-leg standing with eyes closed, and maximum walking speed) among community-dwelling middle-aged and older Japanese individuals.

The human body contains taurine equivalent to 0.1% of the body weight, of which over 50% is present in the muscle (28). Taurine improves anaerobic exercise performance, including muscle strength (29–31). However, whereas the previous studies focused on massive exposure to taurine, the present study focused on the daily intake of taurine.

Taurine intake was significantly and positively associated with knee extension muscle strength (Table 3; Figure 2). Participants with high taurine intake had a higher intake of cereals, potatoes, non-green yellow vegetables, mushrooms, and energy (Table 2). This suggests that people with a high taurine intake consume relatively high amounts of food. However, similar results were obtained after adjusting for energy (data not shown). Therefore, we believe that this finding was not simply an effect of food intake.

Knee extension muscle strength is an indicator of lower limb muscle strength, which is directly related to the ability to perform activities of daily living, such as walking and standing. Muscle strength is influenced by the muscle cross-sectional area and fast/slow muscle fiber ratio. Aging leads to a decrease in muscle cross-sectional area and fast-muscle fiber size, leading to muscle weakness (32). Animal studies have shown that taurine depletion leads to reduced muscle monocontractility in murine extensor digitorum longus muscles (mainly fast-twitch muscles) (33). This suggests that taurine modulates muscle force generation by promoting the release and uptake of calcium ions in the sarcoplasmic reticulum. Furthermore, taurine has been reported to increase the rate of calcium ion uptake into the sarcoplasmic reticulum in both types I and II human muscle fibers, whereas a decrease in taurine concentration decreases the rate of calcium accumulation (34). This suggests that taurine delays muscle relaxation and affects muscle function. Long-term taurine feeding to older rats increased taurine in the muscle and improved chloride conductance and threshold currents in taurine-treated muscle. Moreover, mice lacking the taurine transporter have shorter lifespans, increased expression of aging markers, particularly in skeletal muscles, and both histological and functional muscle loss (35). Recently, the relationships between taurine and aging in muscles have been reported, including the finding that tissue taurine depletion accelerated muscle aging and shortened lifespan (36). Taurine depletion predisposes the heart to fibrosis, which leads to cardiac fibrosis upon aging (37). Taurine counteracted aging-associated impingement of skeletal muscle regeneration (38). These results suggest that taurine may contribute to the maintenance of normal muscle function in middle-aged and older adults and that long-term taurine intake may increase taurine levels in muscles, modulate calcium ion release/uptake, and contribute to muscle strength maintenance.

Conversely, there was no observed relationship between taurine intake and the other three physical fitness parameters. Specifically, sit-and-reach (flexibility), one-leg standing with eyes closed (balance), and maximum walking speed (mobility) are affected by various exercise capacities, including neural control, joint range of motion, posture, and more, except for muscle strength. Limited reports exist regarding the association between taurine intake and these fitness components. It is plausible that taurine intake primarily influences muscle strength or endurance among physical fitness abilities.

Incidentally, a provisional taurine content table was prepared for 666 of the 751 foods in five food groups: seaweed, fish and shellfish, meat, eggs, and milk and dairy products. The table includes 197 items that have been cited from existing databases and academic papers or that were newly measured, as well as 469 items supplemented with similar foods’ taurine contents. The estimated taurine intake in this study (mean 207.5 mg/day) was roughly the same amount as that in a report from South Korea (105 mg in males) (39) and the estimated intake from urinary taurine in Japan (100–300 mg in both males and females) (40), which is similar to the estimated amount of taurine obtained from the Japanese diet (225.5 mg for men and 162.6 mg for women) (16). Based on these results, the provisional taurine content table prepared in this study demonstrated no major problems in calculating dietary taurine intake and is considered useful for understanding dietary taurine intake in the Japanese population.

Considering the relevance of the results of this study to human health, approximately 80% of the participants had a taurine intake of less than 300 mg/day. Moreover, approximately 80% of the taurine was consumed through fish and shellfish (data not shown). Japanese seafood intake has been declining annually, and taurine intake is thought to decline accordingly. Taurine intake decreased by approximately 20% from baseline to follow-up (41). The proportion of older adults in Japan is expected to increase, making healthcare for this population a major social issue. Taurine offers numerous health benefits in addition to the muscle strength maintenance benefits revealed in this study. For example, individuals with high 24-h taurine excretion had lower coronary heart disease mortality, BMI, systolic blood pressure, diastolic blood pressure, total cholesterol, and atherogenesis index than those with low taurine excretion (42). Therefore, dietary taurine intake is expected to contribute to the prevention of care needs by maintaining muscle strength and preventing lifestyle diseases. Specifically, among fish and shellfish, the primary sources of taurine intake for a large number of Japanese people were oysters, octopus, and saury. It is important to ensure a daily sufficient taurine intake by actively including shellfish, cephalopods, and fish in our diet to support healthy aging.

Previous studies have reported a relationship between vitamins and physical fitness (43, 44). Secondary exploratory analysis was used to examine the association between vitamin intake and changes in physical fitness. We found that vitamin B_12_ was significantly and positively associated with knee extension muscle strength, and vitamin B_2_, biotin, and folic acid intake correlated with maximum walking speed in both analysis methods (*p* < 0.05; Supplementary Table S2). This result is generally consistent with previous reports. For example, significant positive associations between overall fitness scores and the intake of vitamins B_6_, C, D, E, niacin, and folic acid have been reported (13). Moreover, a possibility that vitamin B_12_ deficiency might be related to sarcopenia in older adults has been reported (45). Furthermore, elastic band resistance training and nutritional supplementation (rich in proteins, vitamin D, B_2_, and B_12_) improved maximum walking speed and 6-min walking test results (46). Thus, a trend similar to that of previous findings on vitamins was observed, supporting the findings of previous studies. These results suggest a plausible relationship between taurine intake and muscle strength. Further epidemiological studies are required to clarify the relationship between taurine intake and muscle strength.

### 4.1 Limitations and strengths of the study

First, the provisional taurine content table applies cited values to 469 similar products out of 666 products, and the estimated intake may deviate slightly from the actual intake because the taurine content in foods varies widely depending on the season and region. Furthermore, this study used 3-day dietary records to estimate nutrient intake; however, it is unclear whether this reflects the habitual dietary intake. In addition, only baseline intake was used to analyze the association between taurine and physical fitness, which did not consider information on fluctuations in intake. These limitations may have affected the results and interpretation of this study. Long-term dietary records and other measurement techniques should be introduced for a more accurate estimation of taurine intake and for understanding the daily intake patterns.

Nevertheless, this study has four strengths. First, it represents the first epidemiological study to demonstrate an association between taurine and muscle strength. Taurine is not listed in the official tables of nutritional composition in Japan (47), and it has been difficult to analyze the dietary intake. This problem was resolved by creating a provisional taurine content table. Second, dietary and nutritional intakes were assessed using 3-day dietary records and photographs. This method is more accurate than that used in the national nutritional survey in Japan. This method may introduce less recall bias than other methods (e.g., dietary recall and self-reported questionnaires). Third, the inclusion of the same individuals who were followed up for more than 8 years provided evidence of a causal association between dietary taurine intake and muscle strength. Finally, we used samples of randomly selected age-and sex-stratified non-institutionalized individuals enrolled from the community. Therefore, these results may be applicable to normal community-dwelling middle-aged and older Japanese individuals.

## 5 Conclusion

Using a provisional taurine content table, we estimated the taurine intake of middle-aged and older adults living in the community. Dietary taurine intake may contribute to the maintenance of knee extension muscle strength over an 8-year period.

## Data availability statement

The datasets generated for this study are available from the corresponding author upon reasonable request.

## Ethics statement

The studies involving humans were approved by the Ethics Committees of the National Institute for Longevity Sciences and Taisho Pharmaceutical Co., Ltd. The studies were conducted in accordance with the local legislation and institutional requirements. The participants provided their written informed consent to participate in this study.

## Author contributions

TD: Conceptualization, Funding acquisition, Investigation, Project administration, Resources, Visualization, Writing – original draft, Writing – review & editing. KK: Conceptualization, Data curation, Formal analysis, Investigation, Methodology, Software, Validation, Writing – review & editing. YO: Data curation, Formal analysis, Investigation, Methodology, Writing – original draft. KF: Conceptualization, Formal analysis, Methodology, Writing – review & editing. YK: Conceptualization, Investigation, Methodology, Writing – review & editing. YN: Conceptualization, Investigation, Methodology, Writing – review & editing. RK: Conceptualization, Investigation, Methodology, Writing – review & editing. RO: Conceptualization, Data curation, Funding acquisition, Investigation, Methodology, Project administration, Resources, Supervision, Writing – review & editing, Writing – original draft.
