# Supplementary material for: Association of taurine intake with changes in physical fitness among community-dwelling middle-aged and older Japanese adults: an 8-year longitudinal study
Source: Front Nutr. 2024 Mar 20;11:1337738. doi: 10.3389/fnut.2024.1337738 (PMC10989742; doi:10.3389/fnut.2024.1337738)
Supplement: Supplementary file 1 [file Data_Sheet_1.docx]

Supplementary Materials

# Supplementary Tables

Supplementary Table 1. Methods used to develop the tentative taurine content tables (by food group)

| Methods | Specific Methods | Seaweed | Fish and shellfish | Meats | Eggs | Milk and dairy products | Total | Ratio |
| --- | --- | --- | --- | --- | --- | --- | --- | --- |
| Citation | Table of Free Amino Acid Content of Foods (Japan Society of Nutrition and Food Science) | 9 | 25 | 17 | 2 | 1 | 54 | 7.2% |
|  | Academic papers | 7 | 85 | 20 |  | 9 | 121 | 16.1% |
|  | New analysis value |  | 5 | 3 |  |  | 8 | 1.1% |
|  | National Standard Food Composition Table (Korean Rural Development Agency) |  | 2 | 4 | 2 | 6 | 14 | 1.9% |
| Complement | Different parts from the same species |  | 3 | 44 | 1 |  | 48 | 6.4% |
|  | Dried food from the same species | 13 | 28 | 2 | 3 | 6 | 52 | 6.9% |
|  | Processed products from the same species |  | 137 | 41 | 11 | 22 | 211 | 28.1% |
|  | Other related species and miscellaneous | 7 | 68 | 78 | 1 | 4 | 158 | 21.0% |
| No content setting | Zero amount of protein in Japanese Standard Tables of Food Composition |  |  |  |  | 4 | 4 | 0.5% |
|  | Foods consumed infrequently (<20 consumers out of approximately 2,200) | 11 | 35 | 35 |  |  | 81 | 10.8% |
| Total | | 47 | 388 | 244 | 20 | 52 | 751 | 100.0% |

Supplementary Table 2 Multivariate analysis of the association between vitamin intake and physical fitness

|  | **Tertiles, mean±SE** | | | | | | | | |  |  |
| --- | --- | --- | --- | --- | --- | --- | --- | --- | --- | --- | --- |
| **Parameter** | **T1 (Low)** | | | **T2 (Middle)** | | | **T3 (High)** | | | **p-value^†^** | **Trend  p-value^‡^** |
|  | Vitamin A intake | | | | | | | | |  |  |
| Knee extension muscle strength [kgf] | 0.55 | ± | 0.94 | 0.35 | ± | 0.96 | 0.64 | ± | 0.95 | 0.286 | 0.823 |
| Sit-and-reach [cm] | −1.97 | ± | 0.56 | −1.73 | ± | 0.57 | −1.78 | ± | 0.57 | 0.701 | 0.497 |
| One-leg standing with eyes closed [s] | −7.30 | ± | 2.40 | −7.78 | ± | 2.43 | −6.95 | ± | 2.43 | 0.396 | 0.760 |
| Maximum walking speed [m/min] | −4.04 | ± | 1.38 | −2.62 | ± | 1.39 | −3.02 | ± | 1.39 | 0.135 | 0.112 |
|  | Vitamin B_1_ intake | | | | | | | | |  |  |
| Knee extension muscle strength [kgf] | 0.30 | ± | 0.95 | 0.71 | ± | 0.94 | 0.53 | ± | 0.95 | 0.298 | 0.578 |
| Sit-and-reach [cm] | −2.24 | ± | 0.57 | −1.62 | ± | 0.56 | −1.75 | ± | 0.57 | 0.050 | 0.075 |
| One-leg standing with eyes closed [s] | −8.20 | ± | 2.43 | −5.93 | ± | 2.41 | −8.04 | ± | 2.41 | 0.885 | 0.884 |
| Maximum walking speed [m/min] | −3.90 | ± | 1.39 | −3.12 | ± | 1.38 | −3.06 | ± | 1.38 | 0.276 | 0.189 |
|  | Vitamin B_2_ intake | | | | | | | | |  |  |
| Knee extension muscle strength [kgf] | 0.05 | ± | 0.94 | 0.78 | ± | 0.96 | 0.91 | ± | 0.94 | 0.072 | 0.038* |
| Sit-and-reach [cm] | −1.93 | ± | 0.56 | −1.96 | ± | 0.57 | −1.64 | ± | 0.57 | 0.189 | 0.287 |
| One-leg standing with eyes closed [s] | −7.04 | ± | 2.40 | −7.02 | ± | 2.44 | −7.88 | ± | 2.42 | 0.620 | 0.454 |
| Maximum walking speed [m/min] | −4.04 | ± | 1.38 | −3.05 | ± | 1.40 | −2.77 | ± | 1.38 | 0.007* | 0.047* |
|  | Vitamin B_6_ intake | | | | | | | | |  |  |
| Knee extension muscle strength [kgf] | 0.20 | ± | 0.95 | 0.74 | ± | 0.95 | 0.75 | ± | 0.95 | 0.231 | 0.215 |
| Sit-and-reach [cm] | −2.07 | ± | 0.56 | −1.76 | ± | 0.57 | −1.58 | ± | 0.57 | 0.054 | 0.085 |
| One-leg standing with eyes closed [s] | −6.89 | ± | 2.41 | −6.69 | ± | 2.42 | −8.54 | ± | 2.43 | 0.352 | 0.157 |
| Maximum walking speed [m/min] | −4.09 | ± | 1.38 | −3.01 | ± | 1.40 | −2.59 | ± | 1.39 | 0.095 | 0.025* |
|  | Vitamin B_12_ intake | | | | | | | | |  |  |
| Knee extension muscle strength [kgf] | −0.01 | ± | 0.94 | 0.63 | ± | 0.96 | 1.04 | ± | 0.94 | 0.016* | 0.012* |
| Sit-and-reach [cm] | −1.89 | ± | 0.56 | −1.97 | ± | 0.57 | −1.73 | ± | 0.56 | 0.291 | 0.565 |
| One-leg standing with eyes closed [s] | −6.36 | ± | 2.41 | −8.37 | ± | 2.44 | −7.71 | ± | 2.41 | 0.546 | 0.227 |
| Maximum walking speed [m/min] | −3.43 | ± | 1.38 | −3.54 | ± | 1.40 | −3.12 | ± | 1.38 | 0.017* | 0.635 |
|  | Vitamin C intake | | | | | | | | |  |  |
| Knee extension muscle strength [kgf] | 0.13 | ± | 0.94 | 0.82 | ± | 0.95 | 0.78 | ± | 0.95 | 0.348 | 0.121 |
| Sit-and-reach [cm] | −1.93 | ± | 0.56 | −1.70 | ± | 0.57 | −1.89 | ± | 0.57 | 0.320 | 0.895 |
| One-leg standing with eyes closed [s] | −6.74 | ± | 2.40 | −7.88 | ± | 2.42 | −7.56 | ± | 2.44 | 0.583 | 0.469 |
| Maximum walking speed [m/min] | −3.82 | ± | 1.38 | −3.26 | ± | 1.39 | −2.71 | ± | 1.40 | 0.115 | 0.086 |
|  | Vitamin D intake | | | | | | | | |  |  |
| Knee extension muscle strength [kgf] | 0.46 | ± | 0.94 | 0.69 | ± | 0.95 | 0.48 | ± | 0.95 | 0.697 | 0.968 |
| Sit-and-reach [cm] | −1.96 | ± | 0.56 | −1.79 | ± | 0.57 | −1.76 | ± | 0.57 | 0.184 | 0.464 |
| One-leg standing with eyes closed [s] | −7.10 | ± | 2.41 | −6.98 | ± | 2.43 | −7.90 | ± | 2.42 | 0.239 | 0.473 |
| Maximum walking speed [m/min] | −3.63 | ± | 1.38 | −3.21 | ± | 1.39 | −3.09 | ± | 1.39 | 0.035* | 0.393 |
|  | Vitamin E intake | | | | | | | | |  |  |
| Knee extension muscle strength [kgf] | 0.53 | ± | 0.95 | 0.49 | ± | 0.95 | 0.58 | ± | 0.95 | 0.482 | 0.896 |
| Sit-and-reach [cm] | −1.99 | ± | 0.56 | −1.86 | ± | 0.57 | −1.67 | ± | 0.57 | 0.423 | 0.230 |
| One-leg standing with eyes closed [s] | −7.45 | ± | 2.41 | −7.24 | ± | 2.43 | −7.26 | ± | 2.42 | 0.698 | 0.868 |
| Maximum walking speed [m/min] | −3.69 | ± | 1.38 | −3.62 | ± | 1.40 | −2.73 | ± | 1.38 | 0.016* | 0.133 |
|  | Vitamin K intake | | | | | | | | |  |  |
| Knee extension muscle strength [kgf] | 0.24 | ± | 0.95 | 0.76 | ± | 0.94 | 0.52 | ± | 0.95 | 0.674 | 0.505 |
| Sit-and-reach [cm] | −2.18 | ± | 0.57 | −1.59 | ± | 0.56 | −1.83 | ± | 0.57 | 0.069 | 0.194 |
| One-leg standing with eyes closed [s] | −6.97 | ± | 2.43 | −7.87 | ± | 2.40 | −6.97 | ± | 2.43 | 0.533 | 0.999 |
| Maximum walking speed [m/min] | −3.78 | ± | 1.39 | −2.85 | ± | 1.38 | −3.51 | ± | 1.40 | 0.983 | 0.668 |
|  | Biotin intake | | | | | | | | |  |  |
| Knee extension muscle strength [kgf] | 0.73 | ± | 0.95 | 0.40 | ± | 0.95 | 0.48 | ± | 0.94 | 0.660 | 0.551 |
| Sit-and-reach [cm] | −2.09 | ± | 0.57 | −1.73 | ± | 0.57 | −1.72 | ± | 0.56 | 0.227 | 0.162 |
| One-leg standing with eyes closed [s] | −7.13 | ± | 2.43 | −7.17 | ± | 2.45 | −7.54 | ± | 2.39 | 0.872 | 0.711 |
| Maximum walking speed [m/min] | −4.11 | ± | 1.39 | −3.39 | ± | 1.40 | −2.71 | ± | 1.37 | 0.012* | 0.028* |
|  | Folic acid intake | | | | | | | | |  |  |
| Knee extension muscle strength [kgf] | 0.54 | ± | 0.95 | 0.22 | ± | 0.94 | 1.10 | ± | 0.96 | 0.086 | 0.195 |
| Sit-and-reach [cm] | −2.15 | ± | 0.57 | −1.72 | ± | 0.56 | −1.63 | ± | 0.57 | 0.049* | 0.065 |
| One-leg standing with eyes closed [s] | −6.40 | ± | 2.43 | −7.54 | ± | 2.39 | −8.08 | ± | 2.44 | 0.094 | 0.145 |
| Maximum walking speed [m/min] | −3.82 | ± | 1.39 | −3.47 | ± | 1.37 | −2.48 | ± | 1.40 | 0.003* | 0.042* |
|  | Niacin intake | | | | | | | | |  |  |
| Knee extension muscle strength [kgf] | 0.33 | ± | 0.96 | 0.48 | ± | 0.96 | 0.70 | ± | 0.94 | 0.438 | 0.398 |
| Sit-and-reach [cm] | −1.96 | ± | 0.57 | −1.91 | ± | 0.57 | −1.73 | ± | 0.56 | 0.108 | 0.429 |
| One-leg standing with eyes closed [s] | −6.95 | ± | 2.44 | −7.39 | ± | 2.44 | −7.52 | ± | 2.39 | 0.636 | 0.622 |
| Maximum walking speed [m/min] | −4.54 | ± | 1.40 | −2.67 | ± | 1.40 | −2.90 | ± | 1.37 | 0.110 | 0.015* |
|  | Pantothenic acid intake | | | | | | | | |  |  |
| Knee extension muscle strength [kgf] | 0.21 | ± | 0.95 | 0.59 | ± | 0.94 | 0.85 | ± | 0.96 | 0.127 | 0.140 |
| Sit-and-reach [cm] | −2.08 | ± | 0.56 | −1.84 | ± | 0.56 | −1.51 | ± | 0.57 | 0.144 | 0.042* |
| One-leg standing with eyes closed [s] | −7.71 | ± | 2.42 | −6.72 | ± | 2.40 | −7.81 | ± | 2.45 | 0.994 | 0.928 |
| Maximum walking speed [m/min] | −3.74 | ± | 1.39 | −3.37 | ± | 1.38 | −2.73 | ± | 1.40 | 0.146 | 0.126 |

SAS/STAT 9.3_M1 (SAS Institute Japan Ltd.) was used for the statistical analysis. The general linear model (GLM) and trend test were used to assess the association between the baseline dietary intake of vitamins (vitamin A, B_1_, B_2_, B_6_, B_12_, C, D, E, K, niacin, pantothenic acid, biotin, and folic acid) and the change (Baseline to Follow-up) in the four fitness indices (Knee extension muscle strength, sit-and-reach, closed-eye one-leg standing, and maximum walking speed). Trend associations were assessed by utilizing dummy variables (−1, 0, and 1) assigned to dietary vitamin intake tertiles. In the GLM, baseline dietary vitamin intake was included as a covariate, and in the trend test, group (tertile of dietary vitamin intake) was added as a covariate. The number of participants was as follows: Knee extension muscle strength: n=1,254; sit-and-reach: n=1,426; closed-eyed one-legged standing: n=1,413; maximum walking speed: n=1,385.

GLM: general linear model used; SE, standard error.

^‡^ Trend test: The general linear model was used, assigning dummy variables (−1, 0, and 1) to the tertiles of each dietary intake.

* Significance level at *p*<0.05.

Adjusted variables: baseline sex, age, weight, height, smoking status, self-rated health, education, clinical history of hypertension, heart disease, stroke, dyslipidemia, diabetes, and depressive symptoms. GLM + baseline dietary intake. Trend test: + group (tertile of dietary intake).

The change in each dietary intake was calculated as: “Follow-up" − “Baseline.”
